# Supplementary material for: Systematic and Narrative Review of the Mediating Role of Personal Relationships Between Mental Health and Nutrition
Source: Nutrients. 2025 Jul 14;17(14):2318. doi: 10.3390/nu17142318 (PMC12300011; doi:10.3390/nu17142318)
Supplement: Supplementary file 1 [file nutrients-17-02318-s001.zip › Complementary material_ bias study according to JBI. Nutrients.pdf]

Complementary material: bias study according to JBI

Type and number of articles

- Analytical Cross Sectional Studies 7
- Systematic Reviews 4
- Textual Evidence: Policy 3
- Cohort Studies 3
- Randomized Controlled Trials 3
- Textual Evidence: Narrative 2
- Quasi-Experimental Studies 1

Artículo	Tipo	Justificación
Choedon T et al. (2023). Integrating nutrition and mental health screening... Int J Gynaecol Obstet.	Textual Evidence: Policy	Presents proposals to integrate nutrition and mental health protocols into public programs; does not report original empirical research with directly collected data.
Kiecolt-Glaser JK (2010). Stress, food, and inflammation... Psychosom Med.	Textual Evidence: Narrative	Narrative review article discussing interactions among stress, diet, and inflammation; not a systematic review or empirical study.
Young SL et al. (2021). Perspective: The Importance of Water Security... Adv Nutr.	Textual Evidence: Policy	Position paper offering policy recommendations and a conceptual review, without presenting original empirical data.
Chrysafi M et al. (2024). The Potential Effects of the Ketogenic Diet... Nutrients.	Systematic Reviews	Narrative systematic review compiling and analyzing animal and clinical studies on the ketogenic diet and psychiatric disorders.
Bradley T et al. (2022). Systematic review of lifestyle interventions... Syst Rev.	Systematic Reviews	Systematic review with meta-analysis on lifestyle interventions for individuals with mental disorders.
Lukito W et al. (2019). Maternal contributors to intergenerational nutrition... Asia Pac J Clin Nutr.	Cohort Studies	Retrospective analysis of a cohort initiated in 1988 (Tanjungsari Cohort Study), with intergenerational follow-up.
Gichuru W et al. (2019). Is microfinance associated with changes... BMJ Open.	Systematic Reviews	Systematic review and meta-analysis on microfinance and child health/women's nutrition; clear methodology and results.
Ramírez-Luzuriaga MJ et al. (2021). Influence of enhanced nutrition and psychosocial stimulation... Soc Sci Med.	Cohort Studies	Longitudinal study with structural equation modeling of a Guatemalan child cohort followed into adulthood.
Stahacz C et al. (2024). The impact of food aid interventions... Public Health Nutr.	Systematic Reviews	Systematic review on food aid interventions in developed countries and their effects on mental health and diet.
Aktary ML et al. (2020). Impact of a farmers' market nutrition coupon programme... BMJ Open.	Randomized Controlled Trials	Protocol for a randomized controlled trial (RCT) including intervention and control groups to evaluate farmers' market vouchers in low-income populations.

Bacon L et al. (2002). Evaluating a 'non-diet' wellness intervention... Int J Obes Relat Metab Disord.	Randomized Controlled Trials	Randomized controlled clinical trial comparing a non-diet intervention with a traditional diet program; includes pre- and post-intervention measures.
Ellithorpe ME et al. (2023). Problematic video gaming... Psychology of Popular Media.	Analytical Cross Sectional Studies	Cross-sectional study analyzing associations between gaming behavior and health variables; simultaneous data collection and relational analysis.
Rivera LM & Margevich AK (2023). Implicit ethnic–racial self-stereotyping... Stigma and Health.	Analytical Cross Sectional Studies	Cross-sectional study with moderation analysis among psychological, dietary, and physiological variables; non-experimental design.
Agras WS et al. (1996). Maintenance following a very-low-calorie diet... J Consult Clin Psychol.	Randomized Controlled Trials	Randomized clinical trial comparing weight maintenance procedures after a very-low-calorie diet.
Oftedal S et al. (2021). Sleep, diet, activity, and incident poor self-rated health... Health Psychology.	Cohort Studies	Prospective study based on an Australian population cohort, analyzing health behaviors and their future impact on self-rated health.
Holford D et al. (2023). Planning engagement with web resources... J Occup Health Psychol.	Quasi-Experimental Studies	Mixed-methods study with non-random assignment to conditions (plan vs. no plan), comparing group outcomes; not an RCT.
Walker DOH et al. (2024). Social determinants of mental health... J Am Coll Health.	Analytical Cross Sectional Studies	Cross-sectional study using multiple regressions to analyze associations between social determinants (including food insecurity) and mental health in university students.
Becerra MB & Becerra BJ (2020). Psychological Distress among College Students... IJERPH.	Analytical Cross Sectional Studies	Cross-sectional study with multivariate analysis on food insecurity and psychological distress among students; includes gender-based models.
Tsai JHC & Thompson EA (2015). Effects of Social Determinants on Chinese Immigrant Food Service Workers... J Occup Environ Med.	Analytical Cross Sectional Studies	Study using structural equation modeling based on cross-sectional data on social determinants, mental health, and work performance.
Compton MT (2023). Food and Nutrition Insecurity... Psychiatr Serv.	Textual Evidence: Policy	Professional opinion article calling for political action from the field of psychiatry regarding food insecurity; no original empirical data presented.
Kim D (2021). Financial hardship and social assistance... SSM Popul Health.	Analytical Cross Sectional Studies	Cross-sectional study based on national survey data with multivariate analysis on financial hardship and its relationship with mental health and food insecurity.
Atuoye KN & Luginaah I (2017). Food as a social determinant of mental health... Soc Sci Med.	Analytical Cross Sectional Studies	Cross-sectional study in Ghana using OLS analysis of data on food insecurity and psychological distress among household heads.
Compton MT (2014). Food insecurity as a social determinant of mental health... Psychiatr Ann.	Textual Evidence: Narrative	Argumentative article based on secondary sources reflecting on food insecurity as a mental health factor; does not present empirical research.

1. **Integrating nutrition and mental health screening, risk identification and management in prenatal health programs in India.** Int J Gynaecol Obstet. 2023 Sep;162(3):792-801. doi: 10.1002/ijgo.14728. Epub 2023 Mar 5. Choedon T(1), Sethi V(2), Killeen SL(3), Ganjekar S(4), Satyanarayana V(4), Ghosh S(5), Jacob CM(6)(7), McAuliffe FM(3), Hanson MA(7), Chandra P(4).
Pregnancy is a period of major physiologic, hormonal, and psychological change, increasing the risk of nutritional deficiencies and mental disorders. Mental disorders and malnutrition are associated with adverse pregnancy and child outcomes, with potential long-standing impact. Common mental disorders during pregnancy are more prevalent in low- and middle-income countries (LMICs). In India, studies suggest the prevalence of depression is 9.8%-36.7% and of anxiety is 55.7%. India has seen some promising developments in recent years such as increased coverage of the District Mental Health Program; integration of maternal mental health into the Reproductive and Child Health Program in Kerala; and the Mental Health Care Act 2017. However, mental health screening and management protocols have not yet been established and integrated into routine prenatal care in India. A five-action maternal nutrition algorithm was developed and tested for the Ministry of Health and Family Welfare, aiming to strengthen nutrition services for pregnant women in routine prenatal care facilities. In this paper, we present opportunities and challenges for integration of maternal nutrition and mental health screening and a management protocol at routine prenatal care in India, discuss evidence-based interventions in other LMICs including India, and make recommendations for public healthcare providers.

Choedon, T., Sethi, V., Killeen, S. L., Ganjekar, S., Satyanarayana, V., Ghosh, S., Jacob, C. M., McAuliffe, F. M., Hanson, M. A., & Chandra, P. (2023). Integrating nutrition and mental health screening, risk identification and management in prenatal health programs in India. *International Journal of Gynaecology and Obstetrics, 162*(3), 792–801. https://doi.org/10.1002/ijgo.14728
Type of Study: Textual Evidence – Policy

Criteria	Response
Is the source of the opinion clearly identified?	✓ **Yes**
Does the source of opinion have standing in the field of expertise?	✓ **Yes**
Are the interests of the relevant population the central focus of the opinion?	✓ **Yes**
Is the stated position the result of an analytical process, and is there logic in the opinion expressed?	✓ **Yes**
Is there reference to the extant literature/evidence and any incongruence with it logically defended?	✓ **Yes**
Is the opinion supported by peers?	✓ **Yes**

Include. This article presents a structured policy discussion grounded in national public health developments in India and aligned with international evidence. The authors—recognized experts from diverse fields (nutrition, psychiatry, obstetrics)—clearly define both the problem and the policy context. Their recommendations are analytically derived from comparative evidence across LMICs, particularly India. The logical coherence of the arguments, combined with references to implemented programs and policy gaps, make it a strong and relevant policy-based textual source for inclusion in a systematic review.

2. **Stress, food, and inflammation: psychoneuroimmunology and nutrition at the** Psychosom Med. 2010 May;72(4):365-9. doi: 10.1097/PSY.0b013e3181dbf489. Epub 2010 Apr 21. . cutting edge. Kiecolt-Glaser JK(1).
Inflammation is the common link among the leading causes of death. Mechanistic studies have shown how various dietary components can modulate key pathways to inflammation, including sympathetic activity, oxidative stress, transcription factor nuclear factor-kappaB activation, and proinflammatory cytokine production. Behavioral studies have demonstrated that stressful events and depression can also influence inflammation through these same processes. If the joint contributions of diet and behavior to inflammation were simply additive, they would be important. However, several far more intriguing interactive possibilities are discussed: stress influences food choices; stress can enhance maladaptive metabolic responses to unhealthy meals; and diet can affect mood as well as proinflammatory responses to stressors. Furthermore, because the vagus nerve innervates tissues involved in the digestion, absorption, and metabolism of nutrients, vagal activation can directly and profoundly influence metabolic responses to food, as well as inflammation; in turn, both depression and stress have well-documented negative effects on vagal activation, contributing to the lively interplay between the brain and the gut. As one example, omega-3 fatty acid intake can boost mood and vagal tone, dampen nuclear factor-kappaB activation and responses to endotoxin, and modulate the magnitude of inflammatory responses to stressors. A better understanding of how stressors, negative emotions, and unhealthy meals work together to enhance inflammation will benefit behavioral and nutritional research, as well as the broader biomedical community.

Kiecolt-Glaser, J. K. (2010). Stress, food, and inflammation: psychoneuroimmunology and nutrition at the cutting edge. *Psychosomatic Medicine, 72*(4), 365–369. https://doi.org/10.1097/PSY.0b013e3181dbf489

Type of Study: Textual Evidence – Narrative

**Criteria**	**Response**
Is the source of the opinion clearly identified?	**✓ Yes**
Does the source of opinion have standing in the field of expertise?	**✓ Yes**
Are the interests of the relevant population the central focus of the opinion?	**✓ Yes**

Is the stated position the result of an analytical process, and is there logic in the opinion expressed? ✓ **Yes**

Is there reference to the extant literature/evidence and any incongruence with it logically defended? ✓ **Yes**

Include: This narrative article is authored by Janice Kiecolt-Glaser, a recognized authority in psychoneuroimmunology. The paper logically integrates empirical findings from mechanistic and behavioral studies to propose a coherent and scientifically grounded model on the bidirectional influences among stress, diet, vagal tone, and inflammation. It offers a well-reasoned synthesis of existing literature, presents original interpretive insights, and highlights implications for biomedical research. The work meets all JBI criteria for inclusion as a high-quality narrative opinion piece.

3. **Perspective: The Importance of Water Security for Ensuring Food Security, Good Nutrition, and Well-being.** Adv Nutr. 2021 Jul 30;12(4):1058-1073. doi: 10.1093/advances/nmab003. Young SL(1), Frongillo EA(2), Jamaluddine Z(3)(4), Melgar-Quiñonez H(5), Pérez-Escamilla R(6), Ringler C(7), Rosinger AY(8).
Water security is a powerful concept that is still in its early days in the field of nutrition. Given the prevalence and severity of water issues and the many interconnections between water and nutrition, we argue that water security deserves attention commensurate with its importance to human nutrition and health. To this end, we first give a brief introduction to water insecurity and discuss its conceptualization in terms of availability, access, use, and stability. We then lay out the empirical grounding for its assessment. Parallels to the food-security literature are drawn throughout, both because the concepts are analogous and food security is familiar to the nutrition community. Specifically, we review the evolution of scales to measure water and food security and compare select characteristics. We then review the burgeoning evidence for the causes and consequences of water insecurity and conclude with 4 recommendations: 1) collect more water-insecurity data (i.e., on prevalence, causes, consequences, and intervention impacts); 2) collect better data on water insecurity (i.e., measure it concurrently with food security and other nutritional indicators, measure intrahousehold variation, and establish baseline indicators of both water and nutrition before interventions are implemented); 3) consider food and water issues jointly in policy and practice (e.g., establish linkages and possibilities for joint interventions, recognize the environmental footprint of nutritional guidelines, strengthen the nutrition sensitivity of water-management practices, and use experience-based scales for improving governance and regulation across food and water systems); and 4) make findings easily available so that they can be used by the media, community organizations, and other scientists for advocacy and in governance (e.g., tracking progress towards development goals and holding implementers accountable). As recognition of the importance of water security grows, we hope that so too will the prioritization of water in nutrition research, funding, and policy.

Young, S. L., Frongillo, E. A., Jamaluddine, Z., Melgar-Quiñonez, H., Pérez-Escamilla, R., Ringler, C., & Rosinger, A. Y. (2021). Perspective: The Importance of Water Security for Ensuring Food

Security, Good Nutrition, and Well-being. *Advances in Nutrition, 12*(4), 1058–1073. https://doi.org/10.1093/advances/nmab003
Type of Study: Textual Evidence – Policy

Criteria	Response
Is the source of the opinion clearly identified?	✓ **Yes**
Does the source of opinion have standing in the field of expertise?	✓ **Yes**
Are the interests of the relevant population the central focus of the opinion?	✓ **Yes**
Is the stated position the result of an analytical process, and is there logic in the opinion expressed?	✓ **Yes**
Is there reference to the extant literature/evidence and any incongruence with it logically defended?	✓ **Yes**
Is the opinion supported by peers?	✓ **Yes**

Include: This perspective article is authored by a multidisciplinary team of well-established experts in nutrition, global health, and public policy. It presents a logically constructed and evidence-informed argument for integrating water security into the framework of food and nutrition policy. The authors provide an in-depth analysis of conceptual, empirical, and methodological issues, supported by robust literature and comparative frameworks (e.g., with food security). Their four recommendations are actionable, grounded in evidence, and relevant to both researchers and policymakers, fulfilling all JBI policy appraisal criteria.

4. **The Potential Effects of the Ketogenic Diet in the Prevention and Co-Treatment of Stress, Anxiety, Depression, Schizophrenia, and Bipolar Disorder: From the Basic Research to the Clinical Practice.** Nutrients. 2024 May 21;16(11):1546. doi: 10.3390/nu16111546. Chrysafi M(1), Jacovides C(1)(2), Papadopoulou SK(2), Psara E(1), Vorvolakos T(3), Antonopoulou M(1), Dakanalis A(4)(5), Martin M(1), Voulgaridou G(2), Pritsa A(2), Mentzelou M(1), Giaginis C(1).
BACKGROUND: The ketogenic diet (KD) has been highly developed in the past for the treatment of epileptic pathological states in children and adults. Recently, the current re-emergence in its popularity mainly focuses on the therapy of cardiometabolic diseases. The KD can also have anti-inflammatory and neuroprotective activities which may be applied to the prevention and/or co-treatment of a diverse range of psychiatric disorders.
PURPOSE: This is a comprehensive literature review that intends to critically collect and scrutinize the pre-existing research basis and clinical data of the potential advantageous impacts of a KD on stress, anxiety, depression, schizophrenia and bipolar disorder.

METHODS: This literature review was performed to thoroughly represent the existing research in this topic, as well as to find gaps in the international scientific community. In this aspect, we carefully investigated the ultimate scientific web databases, e.g., PubMed, Scopus, and Web of Science, to derive the currently available animal and clinical human surveys by using efficient and representative keywords.
RESULTS: Just in recent years, an increasing amount of animal and clinical human surveys have focused on investigating the possible impacts of the KD in the prevention and co-treatment of depression, anxiety, stress, schizophrenia, and bipolar disorder. Pre-existing basic research with animal studies has consistently demonstrated promising results of the KD, showing a propensity to ameliorate symptoms of depression, anxiety, stress, schizophrenia, and bipolar disorder. However, the translation of these findings to clinical settings presents a more complex issue. The majority of the currently available clinical surveys seem to be moderate, usually not controlled, and have mainly assessed the short-term effects of a KD. In addition, some clinical surveys appear to be characterized by enormous dropout rates and significant absence of compliance measurement, as well as an elevated amount of heterogeneity in their methodological design.
CONCLUSIONS: Although the currently available evidence seems promising, it is highly recommended to accomplish larger, long-term, randomized, double-blind, controlled clinical trials with a prospective design, in order to derive conclusive results as to whether KD could act as a potential preventative factor or even a co-treatment agent against stress, anxiety, depression, schizophrenia, and bipolar disorder. Basic research with animal studies is also recommended to examine the molecular mechanisms of KD against the above psychiatric diseases.

Chrysafi, M., Jacovides, C., Papadopoulou, S. K., Psara, E., Vorvolakos, T., Antonopoulou, M., Dakanalis, A., Martin, M., Voulgaridou, G., Pritsa, A., Mentzelou, M., & Giaginis, C. (2024). The Potential Effects of the Ketogenic Diet in the Prevention and Co-Treatment of Stress, Anxiety, Depression, Schizophrenia, and Bipolar Disorder: From the Basic Research to the Clinical Practice. *Nutrients, 16*(11), 1546. https://doi.org/10.3390/nu16111546
Type of Study: Systematic Review

Criteria	Response
Is the review question clearly and explicitly stated?	✓ **Yes**
Were the inclusion criteria appropriate for the review question?	✓ **Yes**
Was the search strategy appropriate?	✓ **Yes**
Were the sources and resources used to search for studies adequate?	✓ **Yes**

Were the criteria for appraising studies appropriate?	✓ **Unclear** (Not explicitly described)
Was critical appraisal conducted by two or more reviewers independently?	✕ **No** (Not reported)
Were there methods to minimize errors in data extraction?	✕ **No** (Not reported)
Were the methods used to combine studies appropriate?	✓ **Yes** (Narrative synthesis clearly stated)
Was the likelihood of publication bias assessed?	✕ **No**
Were recommendations for policy and/or practice supported by the reported data?	✓ **Yes**
Were the specific directives for new research appropriate?	✓ **Yes**

Included: Although the article presents itself as a comprehensive literature review and shows strengths in its search strategy and clarity of purpose, it does not meet several core methodological criteria for a systematic review. The study fails to report whether critical appraisal of individual studies was performed, whether more than one reviewer was involved in appraisal and data extraction, and whether publication bias was assessed. Moreover, despite a structured narrative synthesis, the absence of methodological rigor in appraising and synthesizing the evidence compromises the reliability of the conclusions. Taking in account that the article does not meet JBI standards for inclusion as a systematic review, is included because for our conceptual purpose is enough.

5. **Systematic review of lifestyle interventions to improve weight, physical activity and diet among people with a mental health condition.** Syst Rev. 2022 Sep 9;11(1):198. doi: 10.1186/s13643-022-02067-3. Bradley T(1)(2), Campbell E(3), Dray J(4)(5), Bartlem K(4)(5), Wye P(4), Hanly G(4)(5), Gibson L(4)(5), Fehily C(4)(5), Bailey J(4), Wynne O(4), Colyvas K(4), Bowman J(4)(5).
BACKGROUND: People with a mental health condition experience an elevated risk of chronic disease and greater prevalence of health and behaviours. Lifestyle interventions aim to reduce this risk by modifying health behaviours such as physical activity and diet. Previous reviews exploring the efficacy of such interventions for this group have typically limited inclusion to individuals with severe mental illness (SMI), with a focus of impact on weight. This review assessed the efficacy of lifestyle interventions delivered in community or outpatient settings to people with any mental health condition, on weight, physical activity and diet.

METHODS: Eligible studies were randomised or cluster-randomised controlled trials published between January 1999 and February 2019 aiming to improve weight, physical activity or diet, for people with any mental health condition. Two reviewers independently completed study screening, data extraction and assessment of methodological quality. Primary outcome measures were weight, physical activity and diet. Secondary outcome measures were body mass index (BMI), waist circumference, sedentary behaviour and mental health. Where possible, meta-analyses were conducted. Narrative synthesis using vote counting based on direction of effect was used where studies were not amenable to meta-analysis.
RESULTS: Fifty-seven studies were included (49 SMI only), with 46 contributing to meta-analyses. Meta-analyses revealed significant (< 0.05) effect of interventions on mean weight loss (-1.42 kg), achieving 5% weight loss (OR 2.48), weight maintenance (-2.05 kg), physical activity (IPAQ MET minutes: 226.82) and daily vegetable serves (0.51), but not on fruit serves (0.01). Significant effects were also seen for secondary outcomes of BMI (-0.48 units) and waist circumference (-0.87cm), but not mental health (depression: SMD -0.03; anxiety: SMD -0.49; severity of psychological symptoms: SMD 0.72). Studies reporting sedentary behaviour were not able to be meta-analysed. Most trials had high risk of bias, quality of evidence for weight and physical activity were moderate, while quality of evidence for diet was low. CONCLUSION: Lifestyle interventions delivered to people with a mental health condition made statistically significant improvements to weight, BMI, waist circumference, vegetable serves and physical activity. Further high-quality trials with greater consistency in measurement and reporting of outcomes are needed to better understand the impact of lifestyle interventions on physical activity, diet, sedentary behaviour and mental health and to understand impact on subgroups.

Bradley, T., Campbell, E., Dray, J., Bartlem, K., Wye, P., Hanly, G., Gibson, L., Fehily, C., Bailey, J., Wynne, O., Colyvas, K., & Bowman, J. (2022). Systematic review of lifestyle interventions to improve weight, physical activity and diet among people with a mental health condition. *Systematic Reviews, 11*(1), 198. https://doi.org/10.1186/s13643-022-02067-3
Type of Study: Systematic Review

Criteria	Response
Is the review question clearly and explicitly stated?	**✓ Yes**
Were the inclusion criteria appropriate for the review question?	**✓ Yes**
Was the search strategy appropriate?	**✓ Yes**
Were the sources and resources used to search for studies adequate?	**✓ Yes**
Were the criteria for appraising studies appropriate?	**✓ Yes**

Was critical appraisal conducted by two or more reviewers independently?	✓ **Yes**
Were there methods to minimize errors in data extraction?	✓ **Yes**
Were the methods used to combine studies appropriate?	✓ **Yes**
Was the likelihood of publication bias assessed?	✓ **Yes**
Were recommendations for policy and/or practice supported by the reported data?	✓ **Yes**
Were the specific directives for new research appropriate?	✓ **Yes**

Include: This article meets all JBI criteria for a high-quality systematic review. The research question is clearly defined, and the inclusion criteria are relevant and rigorously applied. The authors conducted a comprehensive search across multiple databases and used appropriate methods for critical appraisal, data extraction, and synthesis (including both meta-analyses and narrative synthesis). Bias was assessed, and conclusions were transparently linked to the data. The review offers valuable, evidence-based insights into the effectiveness of lifestyle interventions for individuals with mental health conditions, and makes informed recommendations for future research.

6. **Maternal contributors to intergenerational nutrition, health, and well-being: revisiting the Tanjungsari Cohort Study for effective policy and action in Indonesia** Asia Pac J Clin Nutr. 2019;28(Suppl 1):S1-S16. doi: 10.6133/apjcn.201901_28(S1).0001. Lukito W(1), Wibowo L(2), Wahlqvist ML(3)(4)(5)(6).
Perinatal and maternal mortalities in Java became of concern in the 1980s. Since some 90% of births took place at home, the Tanjungsari (TS) district of West Java was identfied as a locality where community-based risk management strategy might reduce this health burden. In 1987, traditional birth attendants (TBA) were trained to identify risk factors for unfavourable birth outcomes. From January 1st 1988 to December 1989, some 4,000 pregnant women in TS were followed and assigned either a trained or untrained TBA. In the first year, early neonatal, and maternal mortality rates (MMR) (32.9 per 1000 and 170 per 100,000 deliveries respectively) were reduced, but not sustained in the second year. Nationally, MMR was 446 in 2009 and 126 in 2015. Although possible to improve health worker performance, and community engagement, the most likely explanation for benefit attrition is that people and material resources 'downstream' of the TBA services were inadequate. Three decades later, Indonesian neonatal and maternal mortality rates of 14 per 1000 and 126 per 100,000 live births in 2015 (globally 16.2 in 2009 and 216 in 2015) according to UNICEF, still demanded improvement, despite more hospital-based births. The original 1988 cohort of women, their children and grandchildren, can now be interrogated for medium to long term health outcomes of nutritional, such as birth weight and growth, and other risk factors. The evolving TS cohort health and nutrition intermediates and endpoints are

instructive. Maternal and early life factors predict adult energy metabolism and cognitive function.

Lukito, W., Wibowo, L., & Wahlqvist, M. L. (2019). Maternal contributors to intergenerational nutrition, health, and well-being: revisiting the Tanjungsari Cohort Study for effective policy and action in Indonesia. *Asia Pacific Journal of Clinical Nutrition, 28*(Suppl 1), S1–S16. https://doi.org/10.6133/apjcn.201901_28(S1).0001

Type of Study: Cohort Study

Criteria	Response
Were the two groups similar and recruited from the same population?	✓ **Yes**
Were the exposures measured similarly to assign people to both exposed and unexposed groups?	✓ **Yes**
Was the exposure measured in a valid and reliable way?	✓ **Yes**
Were confounding factors identified?	✓ **Yes**
Were strategies to deal with confounding factors stated?	✕ **No**
Were the groups/participants free of the outcome at the start of the study (or at the moment of exposure)?	✓ **Yes**
Were the outcomes measured in a valid and reliable way?	✓ **Yes**
Was the follow up time reported and sufficient to be long enough for outcomes to occur?	✓ **Yes**
Was follow up complete, and if not, were the reasons to loss to follow up described and explored?	✕ **No** (Not addressed)
Were strategies to address incomplete follow up utilized?	✕ **No**
Was appropriate statistical analysis used?	✓ **Yes**

Include: The article presents valuable longitudinal findings from the Tanjungsari Cohort Study, offering insights into maternal and early-life determinants of intergenerational health outcomes.

While it meets key criteria regarding cohort design—such as valid measurement of exposure and outcomes, comparability between groups, and sufficient follow-up period—it fails to address key methodological safeguards, including handling of confounding factors, incomplete follow-up, and loss to follow-up. These omissions limit the reliability and internal validity of the conclusions. Thus, despite the historical importance and scale of the study, it does not fully meet JBI quality standards for inclusion as a robust cohort study. But is useful for the purpose of our article.

7. **Is microfinance associated with changes in women's well-being and children's nutrition? A systematic review and meta-analysis.** BMJ Open. 2019 Jan 28;9(1):e023658. doi: 10.1136/bmjopen-2018-023658. Gichuru W(1), Ojha S(2), Smith S(3), Smyth AR(3), Szatkowski L(1).

BACKGROUND: Microfinance is the provision of savings and small loans services, with no physical collateral. Most recipients are disadvantaged women. The social and health impacts of microfinance have not been comprehensively evaluated.

OBJECTIVE: To explore the impact of microfinance on contraceptive use, female empowerment and children's nutrition in South Asia, Sub-Saharan Africa and Latin America and the Caribbean.

DESIGN: We conducted a systematic search of published and grey literature (1990-2018), with no language restrictions. We conducted meta-analysis, where possible, to calculate pooled ORs. Where studies could not be combined, we described these qualitatively.

DATA SOURCES: EMBASE, MEDLINE, LILACS, CENTRAL and ECONLIT were searched (1990-June 2018).

ELIGIBILITY CRITERIA: We included controlled trials, observational studies and panel data analyses investigating microfinance involving women and children.

DATA EXTRACTION AND SYNTHESIS: Two independent reviewers extracted data and assessed risk of bias. The methodological quality of included studies was assessed using the Cochrane risk-of-bias tool for controlled trials and quasi-experimental studies and a modified Newcastle Ottawa Scale for cross-sectional surveys and analyses of panel data. Meta-analyses were conducted using STATA V.15 (StataCorp).

RESULTS: We included 27 studies. Microfinance was associated with a 64% increase in the number of women using contraceptives (OR 1.64, 95% CI 1.45 to 1.86). We found mixed results for the association between microfinance and intimate partner violence. Some positive changes were noted in female empowerment. Improvements in children's nutrition were noted in three studies.

CONCLUSION: Microfinance has the potential to generate changes in contraceptive use, female empowerment and children's nutrition. It was not possible to compare microfinance models due to the small numbers of studies. More rigorous evidence is needed to evaluate the association between microfinance and social and health outcomes.

Gichuru, W., Ojha, S., Smith, S., Smyth, A. R., & Szatkowski, L. (2019). Is microfinance associated with changes in women's well-being and children's nutrition? A systematic review and meta-analysis. *BMJ Open, 9*(1), e023658. https://doi.org/10.1136/bmjopen-2018-023658

Type of Study: Systematic Review and Meta-analysis

Criteria	Response

Is the review question clearly and explicitly stated?	**✓ Yes**
Were the inclusion criteria appropriate for the review question?	**✓ Yes**
Was the search strategy appropriate?	**✓ Yes**
Were the sources and resources used to search for studies adequate?	**✓ Yes**
Were the criteria for appraising studies appropriate?	**✓ Yes**
Was critical appraisal conducted by two or more reviewers independently?	**✓ Yes**
Were there methods to minimize errors in data extraction?	**✓ Yes**
Were the methods used to combine studies appropriate?	**✓ Yes**
Was the likelihood of publication bias assessed?	**✕ No**
Were recommendations for policy and/or practice supported by the reported data?	**✓ Yes**
Were the specific directives for new research appropriate?	**✓ Yes**

Include: This article demonstrates high methodological rigor as a systematic review and meta-analysis, addressing a clearly defined question on the impacts of microfinance on women's well-being and children's nutrition. The authors used a comprehensive search strategy across multiple databases with no language restrictions, involved dual independent reviewers for selection, appraisal and extraction, and applied standard tools for quality assessment. The synthesis of quantitative data through meta-analysis was appropriate and transparently reported. Although publication bias was not explicitly addressed, the rest of the methodology is sufficiently robust to support inclusion.

8. **Influence of enhanced nutrition and psychosocial stimulation in early childhood on cognitive functioning and psychological well-being in Guatemalan adults.** Soc Sci Med. 2021 Apr;275:113810. doi: 10.1016/j.socscimed.2021.113810. Epub 2021 Mar 3. Ramírez-Luzuriaga MJ(1), DiGirolamo AM(2), Martorell R(3), Ramírez-Zea M(4), Waford R(3), Stein AD(5).
RATIONALE: Early-life nutrition interventions in low and middle-income countries have demonstrated long-term benefits on cognitive skills, however, their influence on socioemotional

outcomes has not been fully explored. Moreover, the mediating processes through which nutrition intervention effects operate and are maintained over time are understudied.
METHODS: We followed-up a cohort of Guatemalan adults who participated as children in a community randomized food-supplementation trial. We examined associations of exposure to nutritional supplementation from conception to age 2 years with executive function (measured using three sub-tests of the NIH Toolbox Cognition Battery) and psychological well-being (measured using two sub-scales of the NIH Toolbox Emotion Battery) at ages 40-57 years (n = 1268). We used structural equation modeling to investigate the mediating role of psychosocial stimulation (measured in childhood using parent reports and ratings of home environments), cognitive ability (measured at ages 26-42 years using standardized tests), and executive function on the association of early-life exposure to nutritional supplementation with adult psychological well-being (n = 1640).
RESULTS: We found positive but inconsistent associations of nutritional supplementation in childhood with executive function and psychological well-being in adulthood. Psychosocial stimulation, cognitive ability, and executive function did not mediate the association of early-life nutritional supplementation with adult psychological well-being. We found strong and positive associations of psychosocial stimulation in childhood with cognitive ability, executive function, and psychological well-being in adulthood.
Moreover, we observed no interaction of exposure to nutritional supplementation and psychosocial stimulation in childhood with cognitive and psychological well-being outcomes in adulthood.
CONCLUSION: Our findings suggest that childhood nutrition interventions have long-lasting effects on cognitive ability and psychological well-being outcomes.

Ramírez-Luzuriaga, M. J., DiGirolamo, A. M., Martorell, R., Ramírez-Zea, M., Waford, R., & Stein, A. D. (2021). Influence of enhanced nutrition and psychosocial stimulation in early childhood on cognitive functioning and psychological well-being in Guatemalan adults. *Social Science & Medicine, 275*, 113810. https://doi.org/10.1016/j.socscimed.2021.113810
Type of Study: Cohort Study

Criteria	Response
1. Were the two groups similar and recruited from the same population?	✓ **Yes**
2. Were the exposures measured similarly to assign people to both exposed and unexposed groups?	✓ **Yes**
3. Was the exposure measured in a valid and reliable way?	✓ **Yes**
4. Were confounding factors identified?	✓ **Yes**
5. Were strategies to deal with confounding factors stated?	✓ **Yes**

6. Were the groups/participants free of the outcome at the start of the study (or at the moment of exposure)? ✓ **Yes**

7. Were the outcomes measured in a valid and reliable way? ✓ **Yes**

8. Was the follow-up time reported and sufficient to be long enough for outcomes to occur? ✓ **Yes**

9. Was follow-up complete, and if not, were the reasons to loss to follow-up described and explored? ✓ **Yes**

10. Were strategies to address incomplete follow-up utilized? ✓ **Yes**

11. Was appropriate statistical analysis used? ✓ **Yes**

Include: This study exemplifies a high-quality cohort design, drawing from a well-characterized population involved in a community-randomized food-supplementation trial in Guatemala. The authors clearly describe exposure timing (from conception to age 2), employ validated tools for outcome measurement in adulthood (NIH Toolbox Cognition and Emotion Batteries), and utilize structural equation modeling to assess mediating effects. Confounders and mediators are thoughtfully considered, and follow-up over decades is well documented with strong retention (n=1268 to 1640). The study's robust longitudinal approach, analytical sophistication, and methodological clarity fully meet the criteria for inclusion.

9 **The impact of food aid interventions on food insecurity, diet quality and mental health in households with children in high-income countries: a systematic review.**. Public Health Nutr. 2024 Oct 4;27(1):e195. doi: 10.1017/S1368980024001769. Stahacz C(1), Alwan NA(1)(2)(3), Taylor E(2)(4), Smith D(2)(4), Ziauddeen N(1)(2).
OBJECTIVE: Households with children accessing food aid in high-income countries are often food insecure. We aimed to review the evidence on food aid interventions in households with children and impact on food insecurity, diet quality and mental health. DESIGN: A systematic search was conducted using Web of Science,
MEDLINE, CINAHL and PsycINFO. Articles published from January 2008 to July 2022 including cross-sectional, cohort and interventional studies in high-income countries were eligible.
SETTING: Food aid is defined as the use of interventions providing free food items by community and/or charitable organisations.
PARTICIPANTS: Two-parent, lone parent or households with a primary caregiver with at least one child ≤ 18 years.
RESULTS: From a total of 10 394 articles, nine were included. Food banks, mobile pantry combined with a free meal for children, backpack provision during school term and food parcel home delivery interventions were evaluated. Food bank models offering additional support such as community programmes, health and social services, cooking classes and free meals for children, client-choice-based models and programmes providing convenient access were associated with

improved food security and diet quality (increased intake of wholegrains, fruit and vegetables). One study reported an improvement in mental health and food bank access at the end of 18 months but not at earlier timepoints and one study reported no change in parents' mental health. CONCLUSIONS: Accessing food aid was linked to improved diet quality and reduced food insecurity in some studies. Allowing clients to choose food items and providing support services were most effective.

Stahacz, C., Alwan, N. A., Taylor, E., Smith, D., & Ziauddeen, N. (2024). The impact of food aid interventions on food insecurity, diet quality and mental health in households with children in high-income countries: a systematic review. *Public Health Nutrition, 27*(1), e195. https://doi.org/10.1017/S1368980024001769

Type of Study: Systematic Review

**Criteria**	**Response**
1. Is the review question clearly and explicitly stated?	✓ **Yes**
2. Were the inclusion criteria appropriate for the review question?	✓ **Yes**
3. Was the search strategy appropriate?	✓ **Yes**
4. Were the sources and resources used to search for studies adequate?	✓ **Yes**
5. Were the criteria for appraising studies appropriate?	✓ **Yes**
6. Was critical appraisal conducted by two or more reviewers independently?	✓ **Yes**
7. Were there methods to minimize errors in data extraction?	✓ **Yes**
8. Were the methods used to combine studies appropriate?	✓ **Yes**
9. Was the likelihood of publication bias assessed?	✕ **No**
10. Were recommendations for policy and/or practice supported by the reported data?	✓ **Yes**
11. Were the specific directives for new research appropriate?	✓ **Yes**

Include: This systematic review addresses a clearly defined research question on the impact of food aid interventions on households with children in high-income countries. The inclusion criteria and search strategy are well detailed, using major databases (Web of Science, MEDLINE, CINAHL, PsycINFO). The authors apply appropriate appraisal tools and report that screening, data extraction, and quality assessment were conducted independently by two reviewers, ensuring reliability.

Although the assessment of publication bias is not explicitly described, the synthesis of findings is methodologically sound. The review draws practical conclusions, highlighting that client-choice models and integrated support services are more effective in improving diet quality and reducing food insecurity. The review also identifies gaps for future research, particularly on mental health outcomes and long-term effects.

The systematic review is relevant, methodologically rigorous, and informative for policy and practice, justifying its inclusion.

10. **Impact of a farmers' market nutrition coupon programme on diet quality and psychosocial well-being among low-income adults: protocol for a randomised controlled trial and a longitudinal qualitative investigation.** BMJ Open. 2020 May 5;10(5):e035143. doi: 10.1136/bmjopen-2019-035143. Aktary ML(1), Caron-Roy S(1), Sajobi T(2), O'Hara H(3), Leblanc P(3), Dunn S(2), McCormack GR(1)(2)(4), Timmins D(2), Ball K(5), Downs S(6), Minaker LM(7), Nykiforuk CI(8), Godley J(9), Milaney K(2), Lashewicz B(2), Fournier B(10), Elliott C(1)(11), Raine KD(8), Prowse RJ(8), Olstad DL(12)(2).

INTRODUCTION: Low-income populations have poorer diet quality and lower psychosocial well-being than their higher-income counterparts. These inequities increase the burden of chronic disease in low-income populations. Farmers' market subsidies may improve diet quality and psychosocial well-being among low-income populations. In Canada, the British Columbia (BC) Farmers' Market Nutrition Coupon Programme (FMNCP) aims to improve dietary patterns and health among low-income participants by providing coupons to purchase healthy foods from farmers' markets. This study will assess the impact of the BC FMNCP on the diet quality and psychosocial well-being of low-income adults and explore mechanisms of programme impacts.

METHODS AND ANALYSIS: In a parallel group randomised controlled trial, low-income adults will be randomised to an FMNCP intervention (n=132) or a no-intervention control group (n=132). The FMNCP group will receive 16 coupon sheets valued at CAD$21/sheet over 10-15 weeks to purchase fruits, vegetables, dairy, meat/poultry/fish, eggs, nuts and herbs at farmers' markets and will be invited to participate in nutrition skill-building activities. Overall diet quality (primary outcome), diet quality subscores, mental well-being, sense of community, food insecurity and malnutrition risk (secondary outcomes) will be assessed at baseline, immediately post-intervention and 16 weeks post-intervention. Dietary intake will be assessed using the Automated Self-Administered 24-hour Dietary Recall. Diet quality will be calculated using the Healthy Eating Index-2015. Repeated measures mixed-effect regression will assess differences in outcomes between groups from baseline to 16 weeks post-intervention. Furthermore, 25-30 participants will partake in semi-structured interviews during and 5 weeks after programme completion to explore participants' experiences with and perceived outcomes from the programme.

ETHICS AND DISSEMINATION: Ethical approval was obtained from the University of Calgary Conjoint Health Research Ethics Board, Rutgers University Ethics and Compliance, and University of Waterloo Office of Research Ethics. Findings will be disseminated through policy briefs, conference presentations and peer-reviewed publications.

Aktary, M. L., Caron-Roy, S., Sajobi, T., et al. (2020). Impact of a farmers' market nutrition coupon programme on diet quality and psychosocial well-being among low-income adults: protocol for a randomised controlled trial and a longitudinal qualitative investigation. *BMJ Open, 10*(5), e035143. https://doi.org/10.1136/bmjopen-2019-035143

Type of Study: Protocol for a Randomised Controlled Trial and Qualitative Study

Criteria	Response
1. Was true randomization used for assignment of participants to treatment groups?	✓ **Yes**
2. Was allocation to treatment groups concealed?	✓ **Yes**
3. Were treatment groups similar at the baseline?	❓ **Not yet** *(protocol stage)*
4. Were participants blind to treatment assignment?	✕ **No**
5. Were those delivering treatment blind to treatment assignment?	✕ **No**
6. Were outcome assessors blind to treatment assignment?	❓ **Unclear**
7. Were treatment groups treated identically other than the intervention of interest?	✓ **Yes**
8. Was follow-up complete and if not, were differences between groups explained?	❓ **Not yet** *(future data)*
9. Were participants analyzed in the groups to which they were randomized (intention-to-treat analysis)?	✓ **Planned**
10. Were outcomes measured in the same way for treatment groups?	✓ **Yes**
11. Were outcomes measured reliably?	✓ **Yes**
12. Was appropriate statistical analysis used?	✓ **Yes**

13. Was the trial design appropriate and any deviations from the standard RCT design accounted for in the conduct? ✓ **Yes**

Included: This is a well-designed protocol for a randomized controlled trial with qualitative elements. Although it cannot provide evidence of effect yet, it demonstrates a clear plan for rigorous evaluation of a real-world food aid intervention. If your review includes *emerging or planned interventions*, or if you wish to highlight gaps in current empirical evidence, this protocol could be cited in support of future research directions.

11. **Evaluating a 'non-diet' wellness intervention for improvement of metabolic fitness, psychological well-being and eating and activity behaviors.** Int J Obes Relat Metab Disord. 2002 Jun;26(6):854-65. doi: 10.1038/sj.ijo.0802012. Bacon L, Keim NL, Van Loan MD, Derricote M, Gale B, Kazaks A, Stern JS.
CONTEXT: Current public health policy recommends weight loss for obese individuals, and encourages energy-restricted diets. Others advocate an alternative, 'non-diet' approach which emphasizes eating in response to physiological cues (eg hunger and satiety) and enhancing body acceptance.
OBJECTIVE: To evaluate the effects of a 'health-centered' non-diet wellness program, and to compare this program to a traditional 'weight loss-centered' diet program.
DESIGN: Six-month, randomized clinical trial.
SETTING: Free-living, general community.
PARTICIPANTS: Obese, Caucasian, female, chronic dieters, ages 30-45 y (n=78).
INTERVENTIONS: Six months of weekly group intervention in a non-diet wellness program or a traditional diet program, followed by 6 months of monthly after-care group support.
OUTCOME MEASURES: Anthropometry (weight, body mass index); metabolic fitness (blood pressure, blood lipids); energy expenditure; eating behavior (restraint, eating disorder pathology); psychology (self-esteem, depression, body image); attrition and attendance; and participant evaluations of treatment helpfulness. Measures obtained at baseline, 3 months, 6 months and 1 y.
RESULTS: (1 y after program initiation): Cognitive restraint increased in the diet group and decreased in the non-diet group. Both groups demonstrated significant improvement in many metabolic fitness, psychological and eating behavior variables. There was high attrition in the diet group (41%), compared to 8% in the non-diet group. Weight significantly decreased in the diet group (5.9+/-6.3 kg) while there was no significant change in the non-diet group (-0.1+/-4.8 kg).
CONCLUSIONS: Over a 1 y period, a diet approach results in weight loss for those who complete the intervention, while a non-diet approach does not. However, a non-diet approach can produce similar improvements in metabolic fitness, psychology and eating behavior, while at the same time effectively minimizing the attrition common in diet programs.

Bacon, L., Keim, N. L., Van Loan, M. D., et al. (2002). Evaluating a 'non-diet' wellness intervention for improvement of metabolic fitness, psychological well-being and eating and activity behaviors. *International Journal of Obesity and Related Metabolic Disorders, 26*(6), 854–865. https://doi.org/10.1038/sj.ijo.0802012
Type of Study: Randomized Controlled Trial (RCT)

Criteria	Response
1. Was true randomization used for assignment of participants to treatment groups?	✓ **Yes**
2. Was allocation to treatment groups concealed?	✓ **Yes**
3. Were treatment groups similar at the baseline?	✓ **Yes**
4. Were participants blind to treatment assignment?	✕ **No** *(not feasible)*
5. Were those delivering treatment blind to treatment assignment?	✕ **No** *(group-based behavioral program)*
6. Were outcome assessors blind to treatment assignment?	❓ **Unclear**
7. Were treatment groups treated identically other than the intervention of interest?	✓ **Yes**
8. Was follow-up complete and if not, were differences between groups explained?	✓ **Yes** *(noted high attrition in diet group)*
9. Were participants analyzed in the groups to which they were randomized (intention-to-treat analysis)?	❓ **Unclear**, likely per-protocol
10. Were outcomes measured in the same way for treatment groups?	✓ **Yes**
11. Were outcomes measured reliably?	✓ **Yes**
12. Was appropriate statistical analysis used?	✓ **Yes**
13. Was the trial design appropriate and any deviations from the standard RCT design accounted for in the conduct?	✓ **Yes**

Include – High relevance for assessing psychosocial and metabolic effects of non-diet and diet interventions. Strengths: Clear RCT design, well-defined interventions, rich outcome set including psychological variables. Limitations: No blinding, some attrition bias, uncertain ITT analysis.

12. Ellithorpe, M. E., Meshi, D., & Tham, S. M. (2023). **Problematic video gaming is associated with poor sleep quality, diet quality, and personal hygiene**. *Psychology of Popular Media, 12*(2), 248–253. https://doi.org/10.1037/ppm0000397
Problematic gamers may focus on game-play so much that they forego regular daily activities in favor of more game time, leading to health impairments. Because problematic gamers often desire to return to the game as quickly as possible, they are known to make myopic choices that favor short-term benefits at the cost of long-term gains. The present research examines certain health behaviors that are likely to suffer from such myopic decision-making: sleep quality, diet quality, and personal hygiene. Although other research has assessed the relationships between gaming behavior and each of these health behaviors separately, they are likely to be intercorrelated—representing a pattern of unhealthy decision-making. To achieve our research goal of understanding how problematic gaming might be associated with these health behaviors, we surveyed a university-based sample (n = 354), including targeted sampling of high-intensity gamers. We assessed problematic gaming's relationship with poorer sleep quality, diet quality, and personal hygiene behaviors. Our results reveal a significant association between problematic gaming and all 3 negative health behaviors. Negative health behaviors associated with problematic gaming may be a potential sign that there is a behavioral addiction issue. Interventions should consider a pattern of such behaviors along with gaming behavior to encourage healthier behavioral choices and game-play. (PsycInfo Database Record (c) 2023 APA, all rights reserved)

Ellithorpe, M. E., Meshi, D., & Tham, S. M. (2023). Problematic video gaming is associated with poor sleep quality, diet quality, and personal hygiene. *Psychology of Popular Media, 12*(2), 248–253. https://doi.org/10.1037/ppm0000397
Type of Study: Analytical Cross-Sectional Study

Criteria	Response
1. Were the criteria for inclusion in the sample clearly defined?	✓ **Yes**
2. Were the study subjects and the setting described in detail?	✓ **Yes**
3. Was the exposure measured in a valid and reliable way?	✓ **Yes** *(validated gaming scales used)*
4. Were objective, standard criteria used for measurement of the condition?	✓ **Yes** *(self-reported health behaviors using established scales)*

5. Were confounding factors identified? | ❓ **Unclear** *(no detailed discussion of confounders or adjustment)*

6. Were strategies to deal with confounding factors stated? | ✕ **No**

7. Were the outcomes measured in a valid and reliable way? | ✓ **Yes**

8. Was appropriate statistical analysis used? | ✓ **Yes**

Include – Relevant to understanding how subjective psychological behaviors (problematic gaming) relate to health-related lifestyle behaviors (sleep, diet, hygiene). Strengths: Use of validated measures; direct relevance to behavioral decision-making and psychological well-being. Limitations: No adjustment for confounders; correlational design.

13. Rivera, L. M., & Margevich, A. K. (2023). **Implicit ethnic–racial self-stereotyping's relation to children's body mass index and diet: The moderating role of self-esteem**. *Stigma and Health, 8*(4), 416–427. https://doi.org/10.1037/sah0000333

Childhood obesity in the United States has disproportionately affected Latinx and Black children. The authors examine this issue by drawing upon implicit social cognition theory and social–psychological models of health and stress to propose and test a relation between negative implicit self-stereotyping and body mass index (BMI) and diet. Furthermore, it was predicted that self-esteem would buffer this relation because it is a psychological resource that functions to protect against stressors like the psychological experience of stigma. The authors recruited a community sample of 9–12-year-old Latinx and Black children and measured individual differences in implicit and explicit associations between the self and group stereotypes, self-esteem, objective BMI, and diet. Consistent with the main hypotheses, strong negative implicit (but not explicit) self-stereotyping was associated with higher levels of body mass indices in the obesity range and less healthy diet, but only among children with low self-esteem. Among children with high self-esteem, these relations were absent. These results held even after controlling for the contribution of parents' BMI, diet, education, and household income. These data are the first to theoretically and empirically link implicit self-stereotyping and self-esteem with physiological risk factors for chronic health conditions. Thus, this research contributes to understanding disparities among stigmatized ethnic–racial children in the United States and beyond. (PsycInfo Database Record (c) 2024 APA, all rights reserved)

Rivera, L. M., & Margevich, A. K. (2023). Implicit ethnic–racial self-stereotyping's relation to children's body mass index and diet: The moderating role of self-esteem. *Stigma and Health, 8*(4), 416–427. https://doi.org/10.1037/sah0000333

Type of Study: Analytical Cross-Sectional Study

Criteria	Response
1. Were the criteria for inclusion in the sample clearly defined?	✓ **Yes**
2. Were the study subjects and the setting described in detail?	✓ **Yes**
3. Was the exposure measured in a valid and reliable way?	✓ **Yes** *(IAT para estereotipos implícitos; escalas validadas de autoestima)*
4. Were objective, standard criteria used for measurement of the condition?	✓ **Yes** *(IMC objetivo y dieta evaluada)*
5. Were confounding factors identified?	✓ **Yes**
6. Were strategies to deal with confounding factors stated?	✓ **Yes** *(análisis de regresión controlando por BMI parental, ingreso, etc.)*
7. Were the outcomes measured in a valid and reliable way?	✓ **Yes**
8. Was appropriate statistical analysis used?	✓ **Yes**

Include: Estudio riguroso que conecta procesos psicológicos (autoestereotipos implícitos, autoestima) con resultados de salud física y comportamental (IMC, dieta) en población infantil sana. Fortalezas: Medidas objetivas y validadas, análisis multivariado, hipótesis bien fundamentada. Limitaciones: Diseño transversal no permite inferencias causales.

14. Agras, W. S., Berkowitz, R. I., Arnow, B. A., Telch, C. F., Marnell, M., Henderson, J., Morris, Y., & Wilfley, D. E. (1996). **Maintenance following a very-low-calorie diet**. *Journal of Consulting and Clinical Psychology, 64*(3), 610–613. https://doi.org/10.1037/0022-006X.64.3.610
The authors posed 2 questions in this randomized study of maintenance procedures in which participants were followed for 15 mo after completion of a very-low-calorie diet: Would stimulus narrowing during the reintroduction of solid food, achieved by the use of prepackaged foods, improve weight losses and the maintenance of those losses as compared with the use of regular food? Would reintroduction of foods dependent on progress in losing or maintaining weight be superior to reintroduction on a time-dependent basis? Neither the stimulus narrowing condition nor the reintroduction procedure enhanced either maximum weight loss or maintenance of those losses. The stimulus narrowing condition appeared to be poorly tolerated; compliance and

attendance were poorer in this condition than in the regular food condition. (PsycINFO Database Record (c) 2016 APA, all rights reserved)

Agras, W. S., Berkowitz, R. I., Arnow, B. A., Telch, C. F., Marnell, M., Henderson, J., Morris, Y., & Wilfley, D. E. (1996). Maintenance following a very-low-calorie diet. *Journal of Consulting and Clinical Psychology, 64*(3), 610–613. https://doi.org/10.1037/0022-006X.64.3.610

Type of Study: Randomized Controlled Trial (RCT)

Criteria	Response
1. Was true randomization used for assignment of participants to treatment groups?	✓ **Yes**
2. Was allocation to treatment groups concealed?	❓ **Unclear** *(no especificado)*
3. Were treatment groups similar at the baseline?	✓ **Yes**
4. Were participants blind to treatment assignment?	✕ **No** *(no aplicable por naturaleza de intervención con alimentos visibles)*
5. Were those delivering treatment blind to treatment assignment?	✕ **No** *(no aplicable)*
6. Were outcome assessors blind to treatment assignment?	❓ **Unclear** *(no se menciona)*
7. Were treatment groups treated identically other than the intervention of interest?	✓ **Yes**
8. Was follow-up complete, and if not, were differences between groups in terms of follow-up adequately described and analyzed?	✓ **Yes** *(pérdida mayor en grupo con estímulo reducido, reportada)*
9. Were participants analyzed in the groups to which they were randomized?	✓ **Yes**
10. Were outcomes measured in the same way for treatment groups?	✓ **Yes**

11. Were outcomes measured reliably?	✓ **Yes**
12. Was appropriate statistical analysis used?	✓ **Yes**
13. Was the trial design appropriate, and any deviations from the standard RCT design accounted for in the conduct and analysis?	✓ **Yes**

Include: Ensayo clínico relevante que examina estrategias psicológicas y conductuales de mantenimiento del peso post-dieta en población sana. Fortalezas: Asignación aleatoria, medida prolongada de resultados, evaluación comparativa de estrategias psicológicas. Limitaciones: No hubo cegamiento posible, y adherencia baja en una de las condiciones.

15. Oftedal, S., Rayward, A. T., Fenton, S., & Duncan, M. J. (2021). **Sleep, diet, activity, and incident poor self-rated health: A population-based cohort study**. *Health Psychology, 40*(4), 252–262. https://doi.org/10.1037/hea0001066

Objective: The prospective relationships between poor sleep health, poor diet quality, and physical inactivity with self-rated health (SRH) are not well described. The aim of this study was to assess individual and joint associations between high-risk health behaviors and incident poor SRH. Method: Participants from the Household Income and Labor Dynamics in Australia longitudinal cohort reporting "good" SRH in 2013 were included (n = 8,853) in 2020 data analysis. Logistic regression was used to assess odds of poor SRH in 2017 associated with (a) individual, (b) count, and (c) unique combinations of high-risk behaviors reported in 2013. Results: In the sample (48% female, M_{age} = 45.2 years, SD = 16.8), poor sleep health (OR = 1.66, 95% CI [1.38, 2.01]), physical inactivity (OR = 1.18, [1.01, 1.38]), and poor diet quality (OR = 1.38, [1.16, 1.65]) were associated with increased odds of poor SRH. Reporting one (OR = 1.76, [1.27, 2.43]), two (OR = 2.16, [1.57, 2.98]), and three (OR = 2.99, [2.02, 4.41]) high-risk behaviors was associated with increased odds of poor SRH. All unique combinations of high-risk behaviors were significantly associated with greater odds of poor SRH, except "poor sleep health only" (prevalence = 1.3%). Odds of poor SRH associated with high-risk behavior combinations ranged from 1.73 (95% CI [1.21, 2.47]) for "physical inactivity only" to 4.11 ([2.66, 6.35]) for "poor sleep health + poor diet quality." Conclusions: Reporting ≥ 1 high-risk behavior was associated with increased odds of poor SRH. The combination of poor sleep health with poor diet quality was associated with the greatest odds of poor SRH. Improving multiple high-risk behaviors in combination may be more effective in preventing decline in SRH than improving any behavior alone. (PsycInfo Database Record (c) 2021 APA, all rights reserved)

Oftedal, S., Rayward, A. T., Fenton, S., & Duncan, M. J. (2021). Sleep, diet, activity, and incident poor self-rated health: A population-based cohort study. *Health Psychology, 40*(4), 252–262. https://doi.org/10.1037/hea0001066

Type of Study: Cohort Study

**Criteria**	**Response**

1. Were the two groups similar and recruited from the same population?	✓ **Yes**
2. Were the exposures measured similarly to assign people to both exposed and unexposed groups?	✓ **Yes**
3. Was the exposure measured in a valid and reliable way?	✓ **Yes**
4. Were confounding factors identified?	✓ **Yes**
5. Were strategies to deal with confounding factors stated?	✓ **Yes** *(ajustes por edad, sexo, etc.)*
6. Were the groups/participants free of the outcome at the start of the study?	✓ **Yes** *(SRH buena al inicio)*
7. Were the outcomes measured in a valid and reliable way?	✓ **Yes**
8. Was the follow-up time reported and sufficient to be long enough for outcomes to occur?	✓ **Yes** *(4 años)*
9. Was follow-up complete, and if not, were differences between groups in terms of follow-up adequately described and analyzed?	✓ **Yes**
10. Were strategies to address incomplete follow-up utilized?	✓ **Yes**
11. Was appropriate statistical analysis used?	✓ **Yes**

Include: A robust cohort study with a well-designed methodology, reliable measures, and rigorous analysis of subjective health factors linked to diet, sleep, and physical activity.

16. Holford, D., Tognon, G., Gladwell, V., Murray, K., Nicoll, M., Knox, A., McCloy, R., & Loaiza, V. (2023). **Planning engagement with web resources to improve diet quality and break up sedentary time for home-working employees: A mixed methods study**. *Journal of Occupational Health Psychology, 28*(4), 224–238. https://doi.org/10.1037/ocp0000356
As home working becomes more common, employers may struggle to provide health promotion interventions that can successfully bridge the gap between employees' intentions to engage in healthier behaviors and actual action. Based on past evidence that action planning can

successfully encourage the adoption of healthier behaviors, this mixed-methods study of a web-based self-help intervention incorporated a randomized planning trial that included quantitative measures of engagement and follow-up qualitative interviews with a subsample of participants. Participants either (a) selected a movement plan for incorporating a series of 2-min exercise videos into their work week to break up sedentary time and a balanced meal plan with recipe cards for a week's lunches and dinners or (b) received access to these resources without a plan. Selecting a movement plan was more effective at increasing engagement with the web resources compared to the no-plan condition. In the follow-up interviews, participants indicated that the plan helped to remind participants to engage with the resources and made it simpler for them to follow the guidance for exercises and meals. Ease of use and being able to fit exercises and meals around work tasks were key factors that facilitated uptake of the resources, while lack of time and worries about how colleagues would perceive them taking breaks to use the resources were barriers to uptake. Participants' self-efficacy was associated with general resource use but not plan adherence. Overall, including plans with online self-help resources could enhance their uptake. (PsycInfo Database Record (c) 2023 APA, all rights reserved)

Holford, D., Tognon, G., Gladwell, V., Murray, K., Nicoll, M., Knox, A., McCloy, R., & Loaiza, V. (2023). Planning engagement with web resources to improve diet quality and break up sedentary time for home-working employees: A mixed methods study. *Journal of Occupational Health Psychology, 28*(4), 224–238. https://doi.org/10.1037/ocp0000356

Type of Study: Mixed Methods Study

**Criteria**	**Response**
1. Was true randomization used for assignment of participants to treatment groups?	✓ **Yes** *(aleatorización explícita para plan vs. sin plan)*
2. Was allocation to treatment groups concealed?	❓ *Unclear (no se especifica ocultación de asignación)*
3. Were treatment groups similar at baseline?	✓ **Yes**
4. Were participants blind to treatment assignment?	❌ **No** *(los participantes sabían si usaban plan o no)*
5. Were those delivering treatment blind to treatment assignment?	❌ **No** *(intervención autoadministrada)*
6. Were outcome assessors blind to treatment assignment?	❓ *Unclear*

7. Were treatment groups treated identically other than the intervention of interest?	✓ **Yes**
8. Was follow-up complete, and if not, were differences explained and analyzed?	✓ **Yes** *(se informa seguimiento y se explican barreras)*
9. Were participants analyzed in the groups to which they were randomized?	✓ **Yes**
10. Were outcomes measured in the same way for treatment groups?	✓ **Yes**
11. Were outcomes measured in a reliable way?	✓ **Yes**
12. Was appropriate statistical analysis used?	✓ **Yes**
13. Was the trial design appropriate, and were deviations from the standard RCT design accounted for?	✓ **Yes** *(diseño mixto bien justificado)*

Include: A valid randomized study evaluating the impact of a psychoeducational and planning intervention on diet and physical activity. Although it is not blinded, its inclusion is justified by its rigorous design and thematic relevance.

17. Dayna O H Walker , Verónica Caridad Rabelo , Oscar Jerome Stewart, Drew N Herbert . (2024) **Social determinants of mental health: the roles of traumatic events, financial strain, housing instability, food insecurity, and commute time.** *J Am Coll Health.* 2024 Dec;72(9):3591-3602. doi: 10.1080/07448481.2023.2185454. Epub 2023 Apr 13. PMID: 37053563.
Objective: To identify social determinants of mental health embedded within college students' living and learning conditions.
Participants: Participants included 215 mostly undergraduate (95%) business students at a diverse, urban west coast public university (48% women; mean age 24).
Methods: Participants completed an online self-report survey measuring affective state, global mental health, anxious and depressive symptoms, as well as social determinants of mental health. Data were analyzed using multiple regression controlling for self-esteem, gender, and race/ethnicity.
Results: Results suggest that higher family income is positively related to mental health, whereas more adverse events (e.g., assault, robbery, serious illness or injury), food insecurity, and commute time are negatively related to mental health. Moderation results indicate a moderate buffering effect of belonging on global mental health among students who experience zero adverse events.

Conclusions: Social determinants can shed light on student's precarious living and learning conditions and resultant effects on students' mental health.
Keywords: adverse life events; commute time; food insecurity; low-income; social determinants of mental health.

Walker, D. O. H., Rabelo, V. C., Stewart, O. J., & Herbert, D. N. (2024). Social determinants of mental health: the roles of traumatic events, financial strain, housing instability, food insecurity, and commute time. *Journal of American College Health, 72*(9), 3591–3602. https://doi.org/10.1080/07448481.2023.2185454

Type of Study: Analytical Cross-Sectional Study

Criteria	Response
1. Were the criteria for inclusion in the sample clearly defined?	✓ **Yes** *(estudiantes universitarios de una universidad urbana)*
2. Were the study subjects and the setting described in detail?	✓ **Yes**
3. Was the exposure measured in a valid and reliable way?	✓ **Yes** *(eventos adversos, ingresos, inseguridad alimentaria, etc.)*
4. Were objective, standard criteria used for measurement of the condition?	✓ **Yes** *(salud mental evaluada con escalas validadas)*
5. Were confounding factors identified?	✓ **Yes** *(género, raza/etnicidad, autoestima)*
6. Were strategies to deal with confounding factors stated?	✓ **Yes** *(regresiones múltiples)*
7. Were the outcomes measured in a valid and reliable way?	✓ **Yes**
8. Was appropriate statistical analysis used?	✓ **Yes**

Include: A well-designed cross-sectional study, with adequate samples and measurements, analyzes the relationship between social determinants and perceived mental health in the university population.

18. Monideepa B. Becerra & Benjamin J. Becerra, 2020. "Psychological Distress among College Students: Role of Food Insecurity and Other Social Determinants of Mental Health," IJERPH, MDPI, vol. 17(11), pages 1-12, June.

Food insecurity is a major social determinant of health and an assessment of how it may impact college students' mental health is imperative, as well as differential associations by self-identified gender. A cross-sectional survey was used among college students of a mid-size minority-serving institution with a final sample size of 302 participants aged 18 years or above. Descriptive, bivariate, and multivariable regressions were conducted, by gender, to assess the role of food insecurity (United States Department of Agriculture (USDA) six-item questionnaire), on mental health outcomes (Kessler-6 scale and self-perception). All the statistical analyses were conducted in SPSS version 24 (IBM, Corp.; Armonk, NY, USA) with an alpha less than 0.05 used to denote significance. Among those with food insecurity, the odds of reporting psychological distress (odds ratio (OR) = 3.645, $p < 0.05$) and an average to very poor self-perceived mental health status (OR = 2.687, $p < 0.05$) were higher compared to their food-secure counterparts, with the results consistent in a gender-specific analysis as well. Compared to men, however, women had higher odds of psychological distress (OR = 2.280, $p < 0.05$), as well as reporting average to very poor self-perceived mental health statuses (OR = 2.700, $p < 0.05$). Among women, any alcohol use in the past 12 months (OR = 2.505, $p < 0.05$) and a low self-perceived physical health status (OR = 3.601, $p < 0.05$) were associated with an average to very poor self-perceived mental health status. Among men, a low perceived physical health status was associated with higher odds of psychological distress (OR = 3.477, $p < 0.05$). The results of our study highlight that food insecurity should be considered a social determinant of mental health wellbeing. In addition, gender-specific trends in mental health highlight the need for targeted interventions for prevention and treatment.

Becerra, M. B., & Becerra, B. J. (2020). Psychological distress among college students: Role of food insecurity and other social determinants of mental health. *International Journal of Environmental Research and Public Health, 17*(11), 1–12. https://doi.org/10.3390/ijerph17113911

Type of Study: Analytical Cross-Sectional Study

**Criteria**	**Response**
1. Were the criteria for inclusion in the sample clearly defined?	✓ **Yes** *(estudiantes ≥18 años de una universidad específica)*
2. Were the study subjects and the setting described in detail?	✓ **Yes**
3. Was the exposure measured in a valid and reliable way?	✓ **Yes** *(inseguridad alimentaria medida con cuestionario USDA-6)*
4. Were objective, standard criteria used for measurement of the condition?	✓ **Yes** *(escala Kessler-6 y autopercepción de salud mental)*

5. Were confounding factors identified?	✓ **Yes** *(género, consumo de alcohol, salud física)*
6. Were strategies to deal with confounding factors stated?	✓ **Yes** *(análisis de regresión multivariable por género)*
7. Were the outcomes measured in a valid and reliable way?	✓ **Yes**
8. Was appropriate statistical analysis used?	✓ **Yes** *(SPSS, regresiones con significancia p<0.05)*

Include: A well-structured cross-sectional study examining the relationship between social determinants, food insecurity, and psychological distress in students, with a focus on gender. Solid methodology and relevant data for your review.

19. Jenny Hsin-Chun Tsai, Elaine Adams Thompson. (2015) **Effects of Social Determinants on Chinese Immigrant Food Service Workers' Work Performance and Injuries: Mental Health as a Mediator.** *J Occup Environ Med*. 2015 Jul;57(7):806-13. doi: 10.1097/JOM.0000000000000477. PMID: 26147549

Objective: The effects of social discrimination, job concerns, and social support on worker mental health and the influence of mental health on occupational health outcomes have been documented intermittently. We propose an integrated, theory-driven model to distinguish the impact of social determinants on work performance and injuries and the mediating effects of mental health problems.

Methods: The US Chinese immigrant food service workers (N = 194) completed a multimeasure interview; we tested the integrated model using structural equation modeling.

Results: Mental health problems, which were associated with decreased work performance and increased injuries, also mediated relationships between job/employment concerns and both work performance and injuries but did not mediate the influences of discrimination and social support.

Conclusions: This research reveals mechanisms by which social determinants influence immigrant worker health, pointing to complementary strategies for reducing occupational health disparities.

Tsai, J. H.-C., & Thompson, E. A. (2015). Effects of social determinants on Chinese immigrant food service workers' work performance and injuries: Mental health as a mediator. *Journal of Occupational and Environmental Medicine, 57*(7), 806–813. https://doi.org/10.1097/JOM.0000000000000477

Type of Study: Analytical Cross-Sectional Study

**Criteria**	**Response**

1. Were the criteria for inclusion in the sample clearly defined?	✓ **Yes** *(trabajadores inmigrantes chinos en el sector restauración en EE. UU.)*
2. Were the study subjects and the setting described in detail?	✓ **Yes**
3. Was the exposure measured in a valid and reliable way?	✓ **Yes** *(discriminación, apoyo social, preocupaciones laborales con instrumentos validados)*
4. Were objective, standard criteria used for measurement of the condition?	✓ **Yes** *(medidas estandarizadas de salud mental, rendimiento y lesiones)*
5. Were confounding factors identified?	✓ **Yes** *(modelo teórico y control de variables en SEM)*
6. Were strategies to deal with confounding factors stated?	✓ **Yes** *(uso de ecuaciones estructurales para modelar mediaciones y relaciones múltiples)*
7. Were the outcomes measured in a valid and reliable way?	✓ **Yes**
8. Was appropriate statistical analysis used?	✓ **Yes** *(modelo de ecuaciones estructurales)*

Include: A solid cross-sectional study with a clear theoretical model and rigorous methodology. Significant for its analysis of the mediating role of mental health in the relationship between social stressors and work outcomes.

20. Michael T Compton (2023) **Food and Nutrition Insecurity: A Social Determinant Hungry for Attention by Mental Health Professionals.** Psychiatr Serv. 2023 Dec 1;74(12):1303-1306. doi: 10.1176/appi.ps.20220511. Epub 2023 May 24. PMID: 37221887.
Food and nutrition security-healthy food being available and households being able to access and use it-is necessary for mental and overall health but is a neglected social determinant of mental health. Mental health professionals should address food and nutrition insecurity by weighing in on federal and state legislation and policies related to food and nutrition; promoting food banks and pantries, "food is medicine" initiatives, and programs offering better opportunities to afford and access whole foods and fresh produce; and addressing food and nutrition insecurity at the individual level in the clinical setting through screening, assessment, treatment, and follow-up.

Keywords: Food insecurity; Food security; Nutrition; Nutrition security; Social determinants of health; Social determinants of mental health.

Compton, M. T. (2023). Food and nutrition insecurity: A social determinant hungry for attention by mental health professionals. *Psychiatric Services, 74*(12), 1303–1306. https://doi.org/10.1176/appi.ps.20220511

Document type: Narrative opinion article / commentary / policy perspective.

Criteria	Response
1. Is the source of the opinion clearly identified?	✓ **Yes** (Michael T. Compton, experto reconocido)
2. Does the source have standing in the field of expertise?	✓ **Yes**
3. Are the interests of the relevant population central to the opinion?	✓ **Yes**
4. Is the focus and context clearly stated?	✓ **Yes**
5. Is there logical reasoning that supports the opinion?	✓ **Yes**
6. Is the argument based on relevant literature or evidence?	✓ **Somewhat** *(referencias indirectas, no datos nuevos)*
7. Is any incongruence with literature addressed?	❌ **No**

Include: Although: It does not present a quantitative or qualitative research design with data collection and analysis; It is not a systematic review, meta-analysis, or primary study; It is a professional opinion article (clinical and public policy perspective). It is included because it is conceptually and thematically very relevant, which is what our article requires.

21. Daniel Kim (2021) **Financial hardship and social assistance as determinants of mental health and food and housing insecurity during the COVID-19 pandemic in the United States.** *SSM Popul Health.* 2021 Jun 29:16:100862. doi: 10.1016/j.ssmph.2021.100862. eCollection 2021 Dec. PMID: 34692973. PMCID: PMC8517203

Background: While social assistance through the U.S. federal CARES Act provided expanded unemployment insurance benefits during the COVID-19 pandemic until the summer of 2020, it is unclear whether social assistance was sufficient in subsequent months to meet everyday spending needs and to curb the adverse health-related sequelae of financial hardship.
Methods: Using multivariable Poisson log-binomial regression and repeated cross-sectional Household Pulse Survey data between September and December 2020 on 91,222 working-aged U.S. adults and 28,842 adult housing renters, this study explored the associations of financial hardship with mental health outcomes and food and housing insecurity after accounting for receipt of social assistance.
Results: Financial hardship rose progressively from September to December 2020, and disproportionately affected Black non-Hispanic and Hispanic Americans and lower-income households. Experiencing considerable financial hardship (vs no hardship) predicted nearly 3-fold higher risks of anxiety and depressive symptoms (e.g., adjusted prevalence ratio, PR of depression = 2.75, 95% CI = 2.54-2.98, $P < .001$), a 23-fold higher risk of food insufficiency (PR = 22.71, 95% CI = 15.62-33.01, $P < .001$), and a 27-fold higher risk of a likely eviction (PR = 27.20, 95% CI = 10.63-69.59, $P < .001$). Across outcomes, these relationships were stronger at each successively higher level of financial hardship (all P values for linear trend <0.001), and more than offset benefits from social assistance.
Conclusions: Even after accounting for social assistance receipt, working-aged adults experiencing financial hardship had markedly greater risks of anxiety and depressive symptoms, food insufficiency, and an anticipated housing eviction. These findings point to the urgent need for direct and sustained cash relief well in excess of current levels of social assistance to mitigate the pandemic's adverse impacts on the well-being of millions of Americans, including vulnerable minority and low-income populations.
Keywords: COVID-19 pandemic; Financial hardship; Food security; Housing security; Mental health; Social assistance.

Kim, D. (2021). Financial hardship and social assistance as determinants of mental health and food and housing insecurity during the COVID-19 pandemic in the United States. *SSM - Population Health, 16*, 100862. https://doi.org/10.1016/j.ssmph.2021.100862

Type Analytical Cross-Sectional Study

1. Were the criteria for inclusion in the sample clearly defined?

Yes. The study explicitly included working-aged U.S. adults (18–64 years old) and a subgroup of renters from the Household Pulse Survey conducted between September and December 2020.

2. Were the study subjects and the setting described in detail?

Yes. The study describes the population (n = 91,222 and 28,842 renters) and the setting (cross-sectional survey during the COVID-19 pandemic in the U.S.).

3. Was the exposure measured in a valid and reliable way?

Yes. Financial hardship was measured through specific survey questions regarding the ability to meet basic spending needs, providing a valid proxy.

4. Were objective, standard criteria used for measurement of the condition?

Yes. Mental health symptoms were assessed using validated screening questions for anxiety and depression. Food and housing insecurity were also measured with structured survey items.

5. Were confounding factors identified?
Yes. The study identified potential confounders such as race/ethnicity, income level, and receipt of social assistance.

6. Were strategies to deal with confounding factors stated?
Yes. The statistical models adjusted for confounding variables using multivariable Poisson log-binomial regression.

7. Were the outcomes measured in a valid and reliable way?
Yes. All outcomes—mental health, food insufficiency, and likelihood of eviction—were measured using established and consistent items in the national survey.

8. Was appropriate statistical analysis used?
Yes. The study used robust Poisson log-binomial regression models and tested for linear trends across levels of financial hardship, which is appropriate for the study design and data structure.

include. This study meets all the JBI methodological quality criteria for analytical cross-sectional studies. It presents rigorous statistical analyses and provides important insights into how financial hardship during the COVID-19 pandemic was associated with mental health problems, food insufficiency, and housing insecurity.

22. Kilian Nasung Atuoye , Isaac Luginaah (2017) **Food as a social determinant of mental health among household heads in the Upper West Region of Ghana** PMID: 28360010 *Soc Sci Med* 2017 May:180:170-180. doi: 10.1016/j.socscimed.2017.03.016. Epub 2017 Mar 10.
According to the World Health Organization, mental distress and related illnesses are becoming leading causes of morbidity and mortality in developing countries. Despite the influence of food insecurity on mental health, empirical understanding of this relationship in sub-Saharan Africa, where incidence of food insecurity is relatively high, is almost non-existent. This study contributes to the literature by examining the association between food insecurity and mental health in the Upper West Region of Ghana. We used Ordinary Least Square (OLS) to analyze cross-sectional data collected on household heads (n = 1438) in 2014 using the Household Food Insecurity Access Scale and the DUKE Health Profile. The results show that heads of severely food insecure ($\beta = 0.934$, $p \le 0.001$) and moderately food secure households ($\beta = 0.759$, $p \le 0.001$) were more likely to report elevated mental distress compared to those from food secure households. We also found that female household heads were more likely to report elevated mental distress ($\beta = 0.164$, $p \le 0.05$) compared to their male counterparts. Our findings suggest the need to improve food security as a strategy targeted at improving overall mental health in the Ghanaian context.
Keywords: Ghana; Household food insecurity; Mental health; Social determinants of health.

Atuoye, K. N., & Luginaah, I. (2017). Food as a social determinant of mental health among household heads in the Upper West Region of Ghana. *Social Science & Medicine, 180*, 170–180. https://doi.org/10.1016/j.socscimed.2017.03.016

Type: Cross Sectional Studies
1. Were the criteria for inclusion in the sample clearly defined?
Yes. The study included household heads (n = 1,438) in the Upper West Region of Ghana, using a

clear sampling strategy from 2014 survey data.

2. Were the study subjects and the setting described in detail?
Yes. The context, geographic region, and demographic characteristics (e.g., gender) of the household heads are clearly outlined.

3. Was the exposure measured in a valid and reliable way?
Yes. Food insecurity was assessed using the well-established Household Food Insecurity Access Scale (HFIAS), appropriate for the cultural and regional context.

4. Were objective, standard criteria used for measurement of the condition?
Yes. Mental distress was measured with the DUKE Health Profile, a validated tool for assessing general health and psychological well-being.

5. Were confounding factors identified?
Yes. The study recognized sociodemographic factors such as gender and household characteristics as potential confounders.

6. Were strategies to deal with confounding factors stated?
Yes. Multivariable Ordinary Least Squares (OLS) regression was used to control for the impact of identified covariates.

7. Were the outcomes measured in a valid and reliable way?
Yes. Mental distress was quantified using a recognized instrument, and the regression outputs were statistically significant and clearly reported.

8. Was appropriate statistical analysis used?
Yes. OLS regression was appropriately applied for cross-sectional data analysis, and significance levels were clearly provided for each result.

I**nclude**. The study meets all JBI quality criteria for analytical cross-sectional studies. It provides high-quality empirical data on the association between food insecurity and mental health in a sub-Saharan African context and offers region-specific insight that enriches the global understanding of social determinants of mental health.

23. Compton, M.T. (2014) **Food insecurity as a social determinant of mental health.** *Psychiatric Annals*. Volume 44, Issue 1, January 2014, Pages 46-51
The term "food security" means that people consistently have access to sufficient, safe, and nutritious food in order to maintain a healthy life. Food security includes both physical and economic access to food that meets dietary needs and food preferences. According to the World Health Organization, food security is a complex, sustainable development issue that is linked to health through nutrition, but also to sustainable economic development, environment, and trade. Furthermore, the World Health Organization notes that food security is built upon three pillars - food availability, food access, and food use - with the latter pillar also involving adequate water and sanitation. Food security is one of several conditions necessary for a population to be well-nourished and physically and mentally healthy. For individuals, families, communities, and entire populations to thrive, they must be sure of their food supply.

Compton, M. T. (2014). Food insecurity as a social determinant of mental health. *Psychiatric Annals, 44*(1), 46–51. https://doi.org/10.3928/00485713-20140108-08

Type: Opinion Papers

1. Is the source of the opinion clearly identified?

Yes. The author, Dr. Michael T. Compton, is a well-established psychiatrist and researcher in public mental health.

2. Does the source of opinion have standing in the field of expertise?

Yes. Compton has published extensively on the intersection of psychiatry and public health, particularly social determinants of mental health.

3. Are the interests of the relevant population the central focus of the opinion?

Yes. The article highlights the importance of food security as a prerequisite for both physical and mental health, focusing especially on vulnerable populations.

4. Is the stated position the result of an analytical process, and is there logic in the opinion expressed?

Yes. The argument logically builds on definitions from the WHO and explores the three pillars of food security in relation to mental health needs.

5. Is there reference to the extant literature?

Yes. The article includes references to WHO guidelines and other scientific discussions on food security and its impact on mental health.

6. Is any incongruence with the literature/sources logically defended?

Not applicable. The article aligns with mainstream understanding and does not contradict established literature.

Include. Although this is a theoretical piece, it meets all JBI criteria for a high-quality opinion paper. It is a valuable contribution that conceptually links food insecurity with mental health and supports the theoretical foundation for related empirical studies in your review.
