# Supplementary material for: Systematic and Narrative Review of the Mediating Role of Personal Relationships Between Mental Health and Nutrition
Source: Nutrients. 2025 Jul 14;17(14):2318. doi: 10.3390/nu17142318 (PMC12300011; doi:10.3390/nu17142318)
Supplement: Supplementary file 1 [file nutrients-17-02318-s001.zip › Complementary material_ Characteristics of the included articles.pdf]

Complementary material: Characteristics of the included articles.

Type and number of articles

- Analytical Cross Sectional Studies 7
- Systematic Reviews 4
- Textual Evidence: Policy 3
- Cohort Studies 3
- Randomized Controlled Trials 3
- Textual Evidence: Narrative 2
- Quasi-Experimental Studies 1

PRISMA Item 17: Table of Included Studies

Study No.	Full Citation	Country / Population	Type of Study / Method	Topic(s) Addressed	Main Findings
41	Kiecolt-Glaser, J. K. (2010). Stress, food, and inflammation: Psychoneuroimmunology and nutrition at the cutting edge. *Psychosomatic Medicine*, 72(4), 365–369. https://doi.org/10.1097/PSY.0b013e3181dbf489	USA / General population	Textual Evidence: Narrative	Nutrition – Mental Health	Links stress-induced inflammation with diet; proposes diet as modifiable risk factor for mental health.
42	Chrysafi, M., et al. (2024). The potential effects of the ketogenic diet in the prevention and co-treatment of stress, anxiety, depression, schizophrenia, and bipolar disorder. *Nutrients*, 16(11), 1546. https://doi.org/10.3390/nu16111546	Not specified	Systematic Reviews	Nutrition – Mental Health	Suggests ketogenic diet may have therapeutic benefits for various psychiatric conditions.

43	Oftedal, S., et al. (2021). Sleep, diet, activity, and incident poor self-rated health: A population-based cohort study. *Health Psychology*, 40(4), 252–261. https://doi.org/10.1037/hea0001061	Australia / Adults	Cohort Studies	Nutrition – Mental Health	Healthy diet and sleep patterns linked to lower risk of poor self-rated health.
44	Bradley, T., et al. (2022). Systematic review of lifestyle interventions... *Systematic Reviews*, 11(1), 198. https://doi.org/10.1186/s13643-022-02035-4	Various / Adults	Systematic Reviews	All three axes	Lifestyle interventions improve diet, weight, and physical activity in people with mental illness.
45	Bacon, L., et al. (2002). Evaluating a 'non-diet' wellness intervention... *International Journal of Obesity*, 26(6), 854–865. https://doi.org/10.1038/sj.ijo.0802002	USA / Adults	Randomized Controlled Trials	All three axes	Wellness approach improved metabolic health, eating behavior, and psychological well-being.
46	Holford, D., et al. (2023). Planning engagement with web resources... *Journal of Occupational Health Psychology*, 28(4), 224–236. https://doi.org/10.1037/ocp0000354	UK / Remote workers	Quasi-Experimental Studies	Nutrition – Mental Health	Diet engagement resources helped reduce sedentary behavior and improved perceived health.
47	Choedon, T., et al. (2023). Integrating nutrition and mental health screening... *Int. J. Gynecol. Obstet.*, 162(3), 792–801. https://doi.org/10.1002/ijgo.14758	India / Pregnant women	Textual Evidence: Policy	All three axes	Combines nutrition and mental health services in prenatal programs to enhance outcomes.
48	Lukito, W., et al. (2019). Maternal contributors to intergenerational nutrition... *Asia Pacific Journal of Clinical Nutrition*, 28(S1), S1–S14. https://doi.org/10.6133/apjcn.201906_28(S1).0001	Indonesia / Maternal-child cohort	Cohort Studies	Nutrition – Mental Health	Maternal mental and nutritional health impact intergenerational well-being.
49	Atuoye, K. N., & Luginaah, I. (2017). Food as a social determinant... *Social Science & Medicine*, 180, 170–180. https://doi.org/10.1016/j.socscimed.2017.03.019	Ghana / Adults	Analytical Cross Sectional Studies	All three axes	Food insecurity linked with poor mental health and weaker social support.
50	Young, S. L., et al. (2021). Perspective: The importance of water... *Advances in Nutrition*, 12(4), 1058–1073. https://doi.org/10.1093/advances/nmab003.	Global Perspective	Textual Evidence: Policy	Nutrition – Mental Health	Water security foundational to achieving good nutrition and mental health.
51	Aktary, M. L., et al. (2020). Impact of a farmers' market nutrition coupon programme... *BMJ Open*, 10(5), e035143. https://doi.org/10.1136/bmjopen-2019-035143	Canada / Low-income adults	Randomized Controlled Trials	All three axes	Improved diet quality and psychosocial well-being expected through market coupon intervention.

52	Becerra, M. B., & Becerra, B. J. (2020). Psychological distress among college students... *IJERPH*, 17(11), 4118. https://doi.org/10.3390/ijerph17114118	USA / Students	Analytical Cross Sectional Studies	All three axes	Food insecurity associated with psychological distress among students.
53	Compton, M. T. (2014). Food insecurity as a social determinant... *Psychiatric Annals*, 44(1), 46–51. https://doi.org/10.3928/00485713-20140108-08	USA / General population	Textual Evidence: Narrative	All three axes	Food insecurity should be prioritized in psychiatric evaluation and care.
54	Compton, M. T. (2023). Food and nutrition insecurity... *Psychiatric Services*, 74(12), 1303–1306. https://doi.org/10.1176/appi.ps.202300124	USA / Mental health context	Textual Evidence: Policy	All three axes	Argues for stronger involvement of psychiatrists in addressing nutrition insecurity.
55	Stahacz, C., et al. (2024). The impact of food aid interventions... *Public Health Nutrition*, 27(1), e195. https://doi.org/10.1017/S1368980024001769	High-income countries / Households	Systematic Reviews	All three axes	Food aid improved mental health and diet quality in households with children.
56	Walker, D. O., et al. (2024). Social determinants of mental health... *Journal of American College Health*, 72(9), 3591–3602. https://doi.org/10.1080/07448481.2023.2219811	USA / Students	Analytical Cross Sectional Studies	All three axes	Food insecurity among key predictors of mental health issues.
57	Rivera, L. M., & Margevich, A. K. (2023). Implicit ethnic–racial self-stereotyping... *Stigma and Health*, 8(4), 416–425. https://doi.org/10.1037/sah0000433	USA / Children	Analytical Cross Sectional Studies	All three axes	Racial self-stereotyping affects diet and BMI; moderated by self-esteem.
58	Ramírez-Luzuriaga, M. J., et al. (2021). Influence of enhanced nutrition... *Social Science & Medicine*, 275, 113810. https://doi.org/10.1016/j.socscimed.2021.113810	Guatemala / Adults	Cohort Studies	All three axes	Early life nutrition and psychosocial interventions enhance adult psychological functioning.
59	Gichuru, W., et al. (2019). Is microfinance associated with changes in women’s well-being... *BMJ Open*, 9(1), e023658. https://doi.org/10.1136/bmjopen-2018-023658	Various LMICs / Women	Systematic Reviews	Mental Health – Interpersonal Relationships	Microfinance associated with improved well-being and children's nutrition.

60	Kim, D. (2021). Financial hardship and social assistance... *SSM - Population Health*, 16, 100862. https://doi.org/10.1016/j.ssmph.2021.100862	USA / General population	Analytical Cross Sectional Studies	All three axes	Financial aid programs reduced mental distress and food insecurity.
61	Ellithorpe, M. E., et al. (2023). Problematic video gaming... *Psychology of Popular Media*, 12(2), 248–258. https://doi.org/10.1037/ppm0000395	USA / Gamers	Analytical Cross Sectional Studies	Nutrition – Interpersonal Relationships	Gaming associated with poor diet, sleep, and hygiene.
62	Agras, W. S., et al. (1996). Maintenance following a very-low-calorie diet... *Journal of Consulting and Clinical Psychology*, 64(3), 610–613. https://doi.org/10.1037/0022-006X.64.3.610	USA / Adults	Randomized Controlled Trials	Nutrition – Interpersonal Relationships	Long-term maintenance difficult after extreme diet without social support.
63	Tsai, J. H. C., & Thompson, E. A. (2015). Effects of social determinants... *Journal of Occupational and Environmental Medicine*, 57(7), 806–813. https://doi.org/10.1097/JOM.0000000000000474	USA / Chinese immigrants	Analytical Cross Sectional Studies	Mental Health – Interpersonal Relationships	Mental health mediates effects of work stress on nutrition and injuries.
