# Supplementary material for: Systematic and Narrative Review of the Mediating Role of Personal Relationships Between Mental Health and Nutrition
Source: Nutrients. 2025 Jul 14;17(14):2318. doi: 10.3390/nu17142318 (PMC12300011; doi:10.3390/nu17142318)
Supplement: Supplementary file 1 [file nutrients-17-02318-s001.zip › nutrients-3722149 - PRISMA Checklist. Nutrients.pdf]

Complementary material: PRISMA Checklist

Section & Topic	Item #	Checklist item	Location where item is reported
**TITLE**	1	Identify the report as a systematic review.	Title page
**ABSTRACT**	2	See the PRISMA 2020 for Abstracts checklist.	Abstract (to be adjusted as per PRISMA Abstract Checklist)
**INTRODUCTION**	3	Describe the rationale for the review.	Section 1. Introduction
	4	Provide an explicit statement of the objectives.	Final paragraph of Section 1
**METHODS**	5	Specify inclusion and exclusion criteria.	Section 2.1. Inclusion and exclusion criteria
	6	Specify all information sources.	Section 2.1. Search strategy
	7	Present full search strategies.	Section 2.1
	8	Specify selection process (e.g., reviewers, independence).	Section 2.1. Study selection process
	9	Specify data collection process (e.g., reviewers, piloting).	Section 2.1. Data extraction
	10	List and define all data items.	Sections 2.1 and 3 (Results)
	11	Describe methods to assess risk of bias in included studies.	Section 2.1. The method used to study the risk of bias was that of the Joanna Briggs Institute (https://jbi.global/critical-appraisal-tools)
	12	Specify effect measures used.	Not applicable because is a qualitative synthesis only described in section 2.1.
	13	Describe synthesis methods and rationale.	Section 2.1 and throughout Section 3. Narrative synthesis structured by a triangular model (mental health–nutrition–relationships)
	14	Describe methods to assess reporting bias.	Section.1.6. The Joanna Briggs Institute (JBI) first requires classifying each article according to its study type (e.g., randomized controlled trial, analytical cross-sectional study), and then applying a specific critical appraisal checklist tailored to that type. This process helps identify potential bias and guides the final decision on whether to include the article in the systematic review.
	15	Describe methods to assess certainty in body of evidence.	Not applicable
**RESULTS**	16	Describe study selection process.	Section 3.1 and PRISMA flow diagram (included in complementary material)
	17	Present characteristics of included studies.	Complementary material and section 3.1.
	18	Present risk of bias for each study.	Section 3.1. and complementary material includes a detailed review of the 23 articles using the JBI method. All 23 articles received the rating of: included.
	19	Present results of individual studies.	Sections 3.1
	20	Present results of syntheses.	Sections 3.1 (structured by the triangular model)
	21	Present assessments of reporting biases.	Section 3.1. As reported in the complementary material, there is no risk of bias.
	22	Present assessments of certainty.	Not applicable
**DISCUSSION**	23	Provide interpretation of results in context.	Sections 3.1 and 4.2
	24	Discuss limitations of evidence.	Section 4.2: “The included studies varied widely in design, populations, and measurement of variables, which limits the comparability and generalizability of findings.”
	25	Discuss limitations of review process.	Section 4.2: “The review process was conducted by two researchers but was not registered in a public database. No formal risk of bias or certainty assessment tools were applied, which may limit reproducibility and confidence in the findings.”
	26	Provide implications for practice, policy, and research.	Section 4.3
**OTHER INFORMATION**	27	State whether review protocol exists and where it can be accessed.	Not registered; stated in final paragraph of Section 2
	28	Describe sources of financial or non-financial support.	Final section: Acknowledgements
	29	Declare competing interests.	Final section: Acknowledgements
	30	Report availability of data, code, and other materials.	Final section: Acknowledgements: “All data are included in this article and its supplementary files.”
