# Supplementary material for: Systematic and Narrative Review of the Mediating Role of Personal Relationships Between Mental Health and Nutrition
Source: Nutrients. 2025 Jul 14;17(14):2318. doi: 10.3390/nu17142318 (PMC12300011; doi:10.3390/nu17142318)
Supplement: Supplementary file 1 [file nutrients-17-02318-s001.zip › nutrients-3722149-supplementary-Search Strategies..pdf]

Complementary Material: Search Strategies.

WEB OF SCIENCE

The Web of Science-specific search strategy, supplementary to common search criteria (e.g., human studies, last 20 years), was:
TI=(nutrient OR nutrition OR diet OR food OR nourishment OR eating OR gastronomy) AND AB=(social OR interpersonal relationships OR socialization OR community OR "social determinants of health") AND TI=("mental health" OR "well-being" OR stress)
All terms were precisely restricted to Title (TI) and Abstract (AB) fields to maintain specificity and replicability.

SCOPUS:
TI (nutrition or diet or food or nourishment or food intake or eating) AND TI mental health AND TI social determinants of health

TI (nutrition OR diet OR food OR nourishment OR "food intake" OR eating) AND TI ("mental health") AND TI ("social determinants of health")
All terms were restricted explicitly to the article title (TI), maximizing topic specificity and replicability. Terms related to nutrition were combined using "OR", and linked to "mental health" and "social determinants of health" with "AND" operators, ensuring precise retrieval of relevant articles.

PUBMED

Pubmed: #1 and #2 and #3. Logica: #1 nutrient or nutrition or diet or gastronomy (titulo); #2 interpersonal relationships or socialization or community (abstract); #3 well-being or mental health or stress (titulo).

The PubMed-specific search strategy, supplementary to common search criteria (e.g., human studies, last 20 years), was:
(#1 AND #2 AND #3)
#1: (nutrient OR nutrition OR diet OR gastronomy) [Title] #2: (interpersonal relationships OR socialization OR community) [Abstract] #3: (well-being OR mental health OR stress) [Title]
Terms were specifically restricted to title and abstract fields, ensuring high precision and replicability.

APA

APA: Title: nutrient OR Title: nutrition OR Title: diet OR Title: gastronomy AND Abstract: social AND PsycInfo Classification: 3360 Health Psychology & Medicine AND Peer-Reviewed Journals only. Result(s): 6. Database(s): APA PsycInfo, APA PsycArticles, APA PsycBooks, APA PsycExtra

The APA-specific search strategy, supplementary to common search criteria (e.g., human studies, last 20 years), was:
Title: (nutrient OR nutrition OR diet OR gastronomy) AND Abstract: social AND PsycInfo Classification: 3360 (Health Psychology & Medicine), limited to Peer-Reviewed Journals.

Databases searched: APA PsycInfo, APA PsycArticles, APA PsycBooks, APA PsycExtra. This targeted approach yielded 6 results, ensuring high specificity and replicability.
